# Scientific and technological mapping of big data architectures in healthcare

**DOI:** 10.3389/fpubh.2026.1775715

**Published:** 2026-07-09

**Authors:** Helder Prado Santos, Methanias Colaço Júnior, Ricardo Valentim, João Paulo Queiroz dos Santos, Marcia Elizabeth Marinho da Silva, Luiz Affonso Henderson Guedes de Oliveira, Guilherme Medeiros Machado, Antonio Higor Freire de Morais, Marianne Dantas Farias Vieira, Deborah Dantas Arruda, Gleyson José Pinheiro Caldeira Silva, Raphael Silva Fontes

**Affiliations:** 1 Laboratory for Technological Innovation in Health (LAIS), Onofre Lopes University Hospital, Federal University of Rio Grande do Norte (UFRN), Natal, Rio Grande do Norte, Brazil; 2 Center for Innovation and Advanced Technology (NAVI), Federal Institute of Rio Grande do Norte (IFRN), Natal, Rio Grande do Norte, Brazil; 3 Postgraduate Program in Computer Science (PROCC), Federal University of Sergipe, São Cristóvão, Brazil; 4 Brazilian Ministry of Health, Brasília, Brazil; 5 ECE Paris Engineering School, Paris, France

**Keywords:** architecture, big data, data lake, health, performance, public health auditing, scalability

## Abstract

**Context:**

The growth of data in healthcare brings both challenges and opportunities. The term Big Data refers to the handling of large volumes of data using advanced techniques and scalable infrastructure. Distributed processing and parallel computing accelerate data processing and necessitate a robust architecture. A Big Data architecture requires distributed systems, security, data storage, processing, analysis, and visualization. Crucially, in a Public Health context, such architectures must also incorporate strict data integrity and epidemiological validation to prevent the rapid processing of biased data.

**Objective:**

This work aims to identify and characterize the approaches, concepts, and software tools used in constructing a general Big Data architecture for healthcare, exploring how these generalized frameworks can be adapted for specific unmet needs, such as health auditing environments, with a focus on performance, scalability, and structural data validity.

**Method:**

A systematic mapping was conducted to identify primary studies in the literature and collect evidence to guide future research and technological adaptations.

**Results:**

A total of 234 articles were analyzed, with Scopus and ACM Digital Library being the most relevant databases. A total of 22 articles were selected after applying inclusion and exclusion criteria and conducting a quality assessment. The most addressed layers were data storage, processing, ingestion, analysis, and visualization. However, a notable gap regarding automated statistical consistency layers was identified, highlighting the need for a post-hoc theoretical proposition of a validation layer. Various software tools were cataloged and grouped by their architectural function. Additionally, comparisons were made between similar software tools, and essential concepts for creating a Big Data architecture were discussed.

**Conclusions:**

The research presented a comprehensive catalog of relevant information for creating a Big Data architecture for healthcare. The study highlights that while foundational building blocks are available, they must be adapted to develop a scalable, high-performance, and analytically rigorous architecture specifically for healthcare auditing, aligning technical infrastructure with methodological validation.

## 1 Introduction

In recent years, there has been an exponential growth in the amount of data produced in healthcare, originating from electronic medical records, patient files, clinical exams, medical images, and genomic data. This data explosion presents both significant challenges and opportunities for healthcare organizations. To manage this massive volume of information, the term Big Data has become increasingly prominent (1).

Big Data refers to the ability to handle large volumes of data that are generated, collected, and stored continuously and at high speed (2). These data are characterized by their variety, velocity, value, volume, and veracity, posing unique challenges in their capture, storage, processing, and analysis.

To extract value and significant insights from this data deluge, advanced techniques and technologies are necessary, such as distributed computing algorithms, parallel processing, machine learning, and real-time data analysis (3). Furthermore, the infrastructure to support Big Data typically involves clusters of high-capacity computers, distributed storage systems, and scalable databases.

Distributed computing plays a crucial role in processing the large data volumes in Big Data. It involves dividing the processing load among multiple nodes in a cluster, allowing complex tasks to be executed concurrently and accelerating overall processing. Parallel computing, in turn, uses multiple processors or processing cores to perform calculations simultaneously, further increasing processing speed (4).

Designing an appropriate architecture to handle Big Data is a complex challenge. It requires the development of distributed and scalable systems capable of processing large amounts of data in real-time without compromising efficiency and fidelity (5). Additionally, it is essential to ensure data security and privacy, as well as to integrate different data sources and types.

From a Public Health and epidemiological perspective, however, treating healthcare data merely as a generic stream of bits is insufficient. Speeding up the processing of biased data is not an achievement; it is a clinical hazard. Therefore, an architecture must ensure scientific validity, methodological adherence, and the veracity of the data being moved.

A Big Data architecture is composed of several layers, such as data ingestion, data storage, data processing, data analysis, and data visualization. Each of these layers plays a fundamental role in the large-scale processing and analysis of data, and there are various options of available software to meet the specific needs of each layer (6).

In this context, this article aims to analyze the state-of-the-art of architectures and software that address the demands of Big Data in healthcare. We present a systematic mapping to identify and characterize the approaches, concepts, and software tools used to build general Big Data architectures. Moreover, it discusses how these generalized frameworks can be adapted and repurposed for specific domains that currently lack tailored solutions, such as healthcare auditing systems, focusing on performance, scalability, and data integrity. Additionally, a bibliometric analysis was conducted based on the temporality, main institutions, origins, and publication venues of the articles.

The remainder of this article is organized as follows: Section 2 describes the planning of the systematic mapping; Section 3 presents the execution of the systematic mapping; Section 4 shows and discusses the results obtained; Section 5 addresses threats to validity; and finally, Section 7 presents the conclusion.

## 2 Planning the systematic mapping

### 2.1 Objective

This mapping aimed to identify and characterize the approaches, concepts, and software used to construct a general *Big Data* architecture that can be adapted and specifically replicated for auditing in a healthcare system, with an emphasis on performance, scalability, and epidemiological data integrity.

### 2.2 Research questions

The research questions were developed to provide a comprehensive view of the field, highlighting fundamental aspects of primary studies (7, 8). They were created based on the PICO model (9), which aims to emphasize the effects of an intervention on a designated population and structure research into four main categories: Population, Intervention, Comparison, and Outcome. These categories, according to (9), can be used to construct research questions of various types. Table 1 shows the PICO model used in this article.

**Table 1:** PICO model for research question compliance.

Acronym	Category	Description
P	Population	Publications that directly address a big data architecture in the healthcare field
I	Intervention	Contexts and approaches for data storage
C	Control	Traditional data storage systems, existing methodologies, alternative big data platforms, performance benchmarks, and similar case studies
O	Outcomes	Tools that bring scalability and performance to the architecture

Therefore, using the PICO model definition, research questions were elaborated based on the guidelines of the Systematic Mapping of Literature observed in (7, 8). They are as follows:

- Q1: What are the layers that compose a Big Data architecture in healthcare?
- Q2: Which software tools are most commonly used in healthcare Big Data architectures?
- Q3: Which architecture presents the best relationship between performance and scalability?
- Q4: In which years were articles in this field most frequently published?
- Q5: What were the most popular publication venues?
- Q6: Which countries have the most publications in this field?

### 2.3 Search and selection strategy

To prepare for the article search, databases were selected from the main periodicals in the Computer Science field. They were:

- SCOPUS < http://www.scopus.com>;
- IEEE Xplore < http://ieeexplore.ieee.org>;
- ACM Digital Library < https://dl.acm.org/>;
- Web of Science < https://www.webofscience.com>;

To execute the search, filters were applied in each database, focusing on the title, abstract, and keywords. Access to the databases was through the CAPES portal (10), using an institutional subscription with no article limit. To conduct the search in digital databases, a string was defined with terms in English and synonyms associated with architectures and software for creating *Big Data* environments. The terms were identified from the studies in the PICO model, defined in Table 1, and subsequently refined and adapted for better use of the string. Table 2 shows the terms before refinement.

**Table 2:** PICO model keywords.

Category	Description
Population	Architecture, big data, health
Intervention	Data lake, data mesh, data warehouse, data mart
Control	-
Intervention	Performance, scalability

From the terms shown above, the following search string was created: • TITLE-ABS-KEY *((“architecture” OR “system design”) AND “big data” AND “health”) AND (“data lake” OR “data mesh” OR “data warehouse” OR “data mart”) AND (“performance” OR “scalability”)*.

It is important to acknowledge a methodological limitation regarding this search strategy. The search string heavily emphasized Computer Science terminology focused on infrastructure, performance, and scalability. By not explicitly including terms related to epidemiological validation, bias detection, or data veracity, this strategy carries the inherent risk of excluding relevant studies focused on clinical data integrity and methodological validation in healthcare architectures.

### 2.4 Source selection criteria

Inclusion and exclusion criteria were established to filter relevant articles for the systematic mapping. After the search using the string shown in Section 2.3, only the studies selected for evaluation were counted, after passing the inclusion and exclusion criteria. The criteria are as follows.

Inclusion Criteria:

1. The article addresses important concepts of a *Big Data* architecture.
2. The article presents a tool that is fundamental for a *Big Data* architecture.
3. The article presents performance or scalability improvements with the use of a software in the deployed architecture.
4. The article proposes an architecture or set of software for mining large volumes of data.
5. The article contains the search string in the title, abstract, or keywords.
6. The article is available online in digital libraries.

Exclusion Criteria:

1. The article is unavailable.
2. The article does not present a tool or *Big Data* architecture.
3. The article is a duplicate.
4. The article is incomplete (e.g., abstract only).
5. The article was published more than 10 years ago.
6. Books.
7. Systematic Reviews.
8. *Surveys*.

### 2.5 Strategy to evaluate article quality

To assess the quality of the articles selected after applying the inclusion and exclusion criteria, a checklist was developed to be filled out after reading each article, as proposed by (7). In this stage, each article was read in full, and each question on the checklist was answered with “Yes”, “Partially”, or “No”. Each answer was assigned a score: 1.0 point for “Yes”, 0.5 points for “Partially”, and 0 points for “No”. Articles that obtained a score equal to or greater than 3.0 points were classified as “Accepted”, while those with a score below 3.0 points were classified as “Rejected”. The quality checklist questions used for the mapping can be seen in Table 3.

**Table 3:** Quality assessment checklist for articles.

1.	Does the article present comparisons between big data tools?
2.	Does the article include any open-source tool that can be replicated?
3.	Does the article provide any important information for building a big data architecture?
4.	Does the article include a scheme of a big data architecture?
5.	Does the article address the topic of big data in the healthcare field?
6.	Does the article address the topic of big data in the auditing field?

### 2.6 Strategy to extract information

To answer the research questions mentioned in Section 2.2, a form was developed to be filled out after reading each article in its entirety. According to (7), data extraction forms should be designed to collect all the necessary information to address the research questions and the study's quality criteria. Table 4 presents the extraction form used in this study.

**Table 4:** Questions related to data extraction from articles.

1.	Which layers of the architecture were presented?
2.	Which software was used?
3.	What comparisons between tools were presented in the study?
4.	What were the fundamental aspects of a big data architecture explained in the study?
5.	What is the publication year of the study?
6.	Does the article address the topic of big data in the auditing field?
7.	Which country published this study?

## 3 Building the systematic map

**Figure 1:**
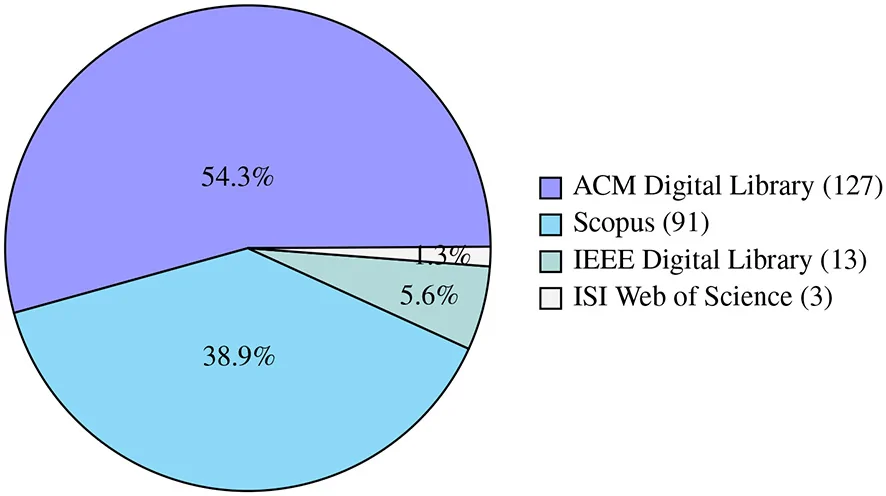
Distribution of articles by publication source (absolute values in parentheses).

The Scopus database (11) was chosen to define and refine the search string, as it indexes some of the main Computer Science periodicals and includes articles from various scientific databases such as IEEE, ACM, Springer, and Elsevier. After being defined, refined, and deemed adequate, the string was adapted for the other search engines used in this study: IEEE Xplore, ACM Digital Library, and Web of Science. A total of 234 studies were found: 127 (54.3%) from ACM Digital Library, 91 (38.9%) from Scopus, 13 (5.6%) from IEEE Digital Library, and 3 (1.3%) from ISI Web of Science. This data is represented in Figure 1.

**Figure 2:**
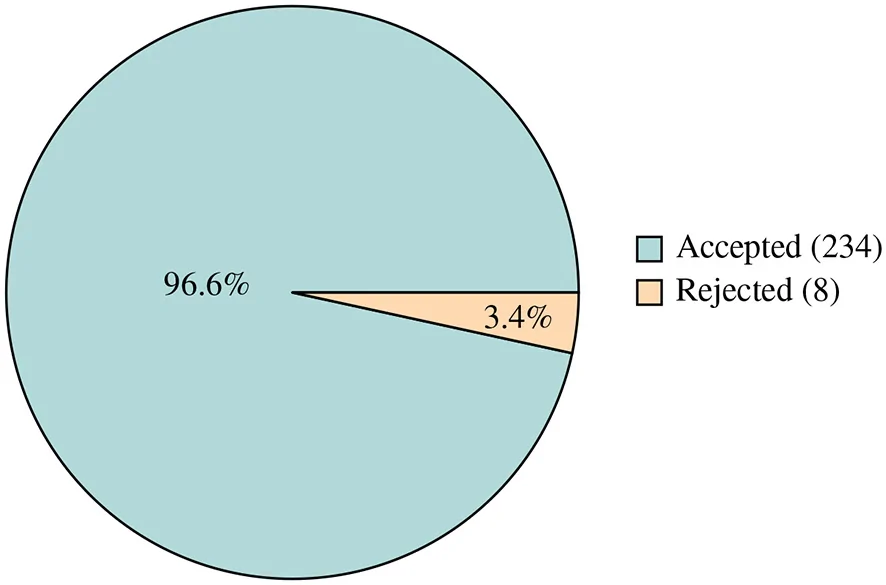
Exclusion of duplicate articles.

After retrieving the articles, the filtering process began by excluding duplicate articles. Each article was classified as Accepted or Rejected. In this stage, 8 (3.4%) of the articles were classified as Rejected and excluded from the mapping. At the end of this stage, 226 (96.6%) were classified as Accepted for the subsequent filtering stages. Figure 2 shows a summary of this stage.

**Figure 3:**
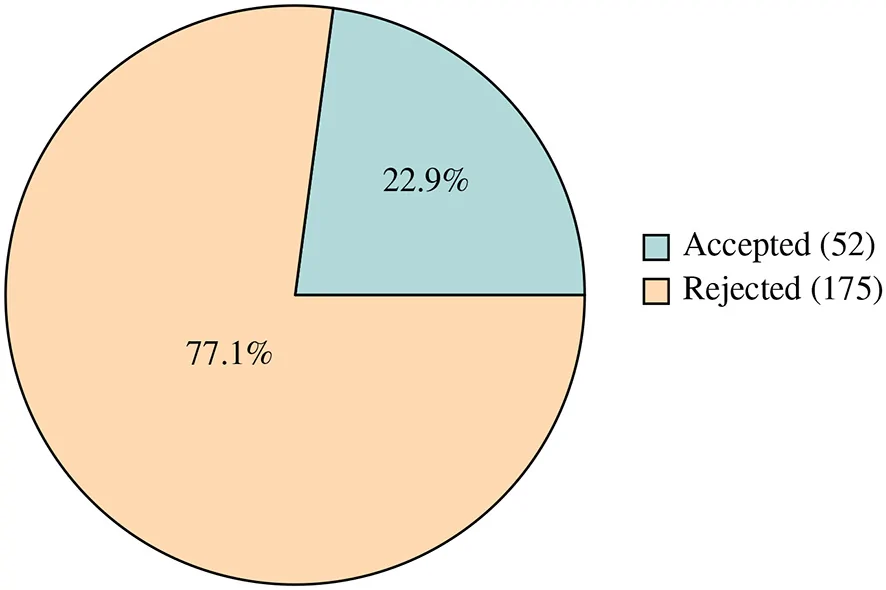
First stage of study selection.

**Figure 4:**
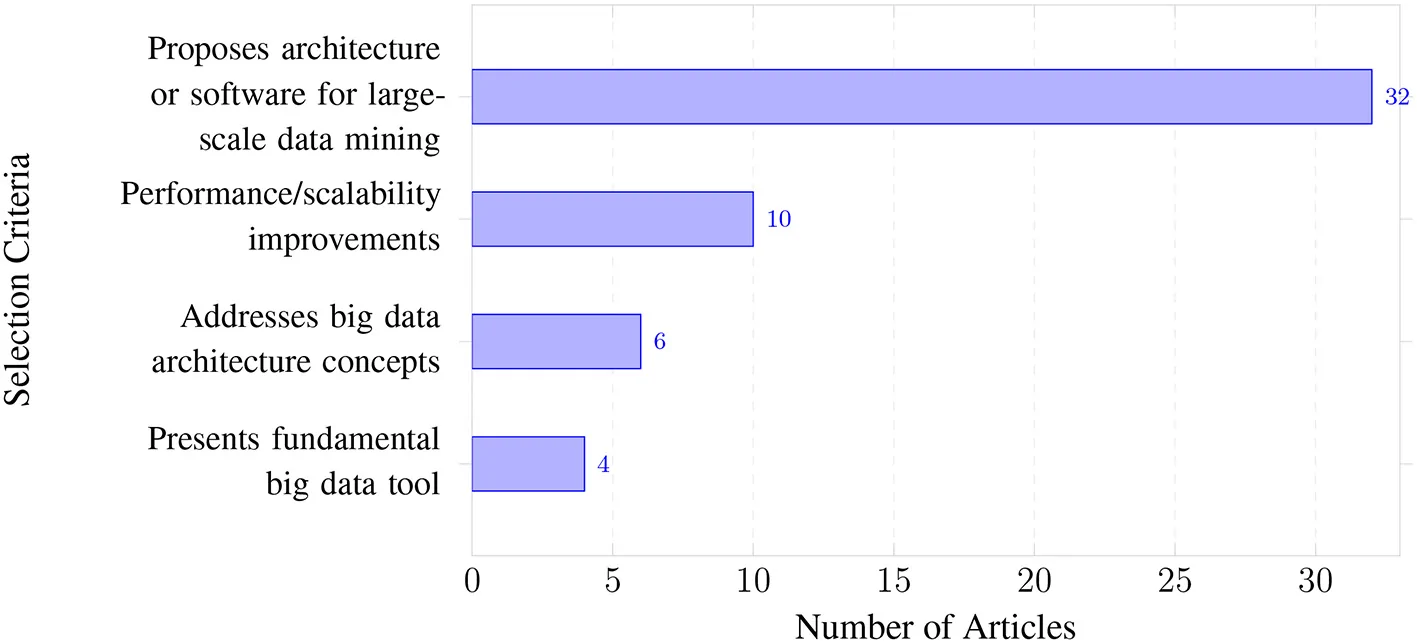
Accepted articles by selection criteria.

**Figure 5:**
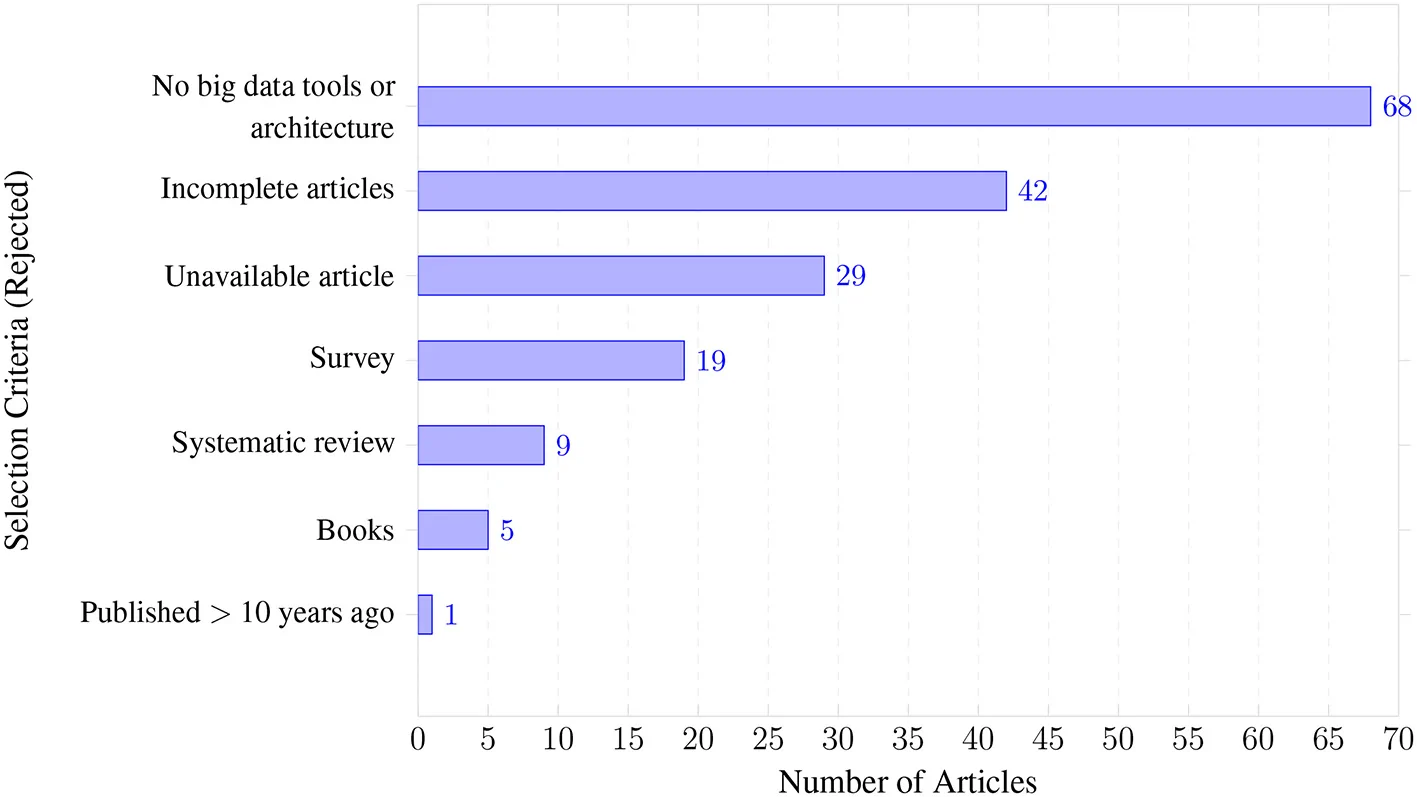
Rejected articles by selection criteria.

After excluding duplicate articles, the first filtering stage began, aimed at verifying whether the articles met the established inclusion or exclusion criteria. In this stage, the articles were superficially evaluated to identify at least one inclusion or exclusion criterion, with the objective of classifying them as Accepted or Rejected. As a result of this stage, 52 articles were selected, representing 22.9% of the total articles filtered after the duplicate exclusion stage. This data is presented in Figure 3. Figure 4 presents the inclusion criteria for each of the Accepted articles, while Figure 5 shows the exclusion criteria for each of the Rejected articles.

**Figure 6:**
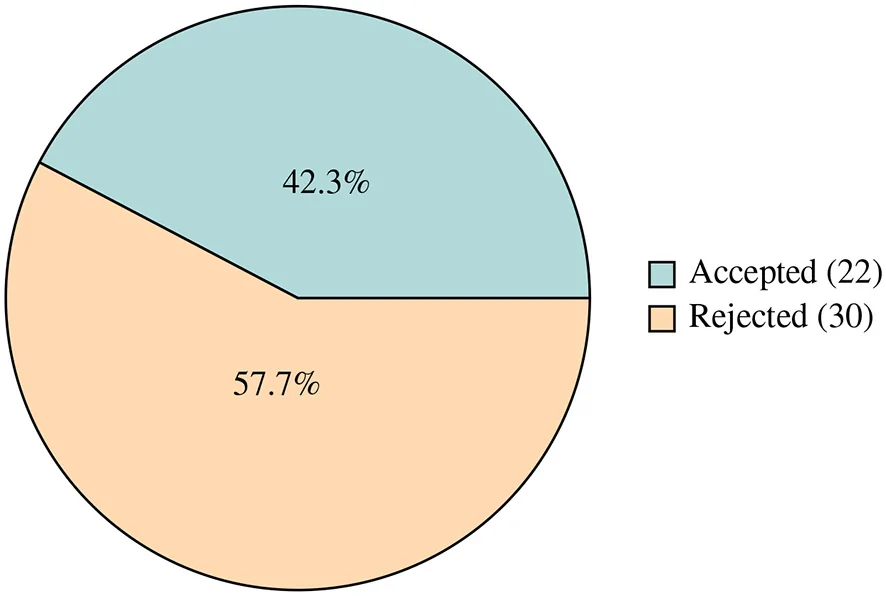
Second stage of study selection.

**Figure 7:**
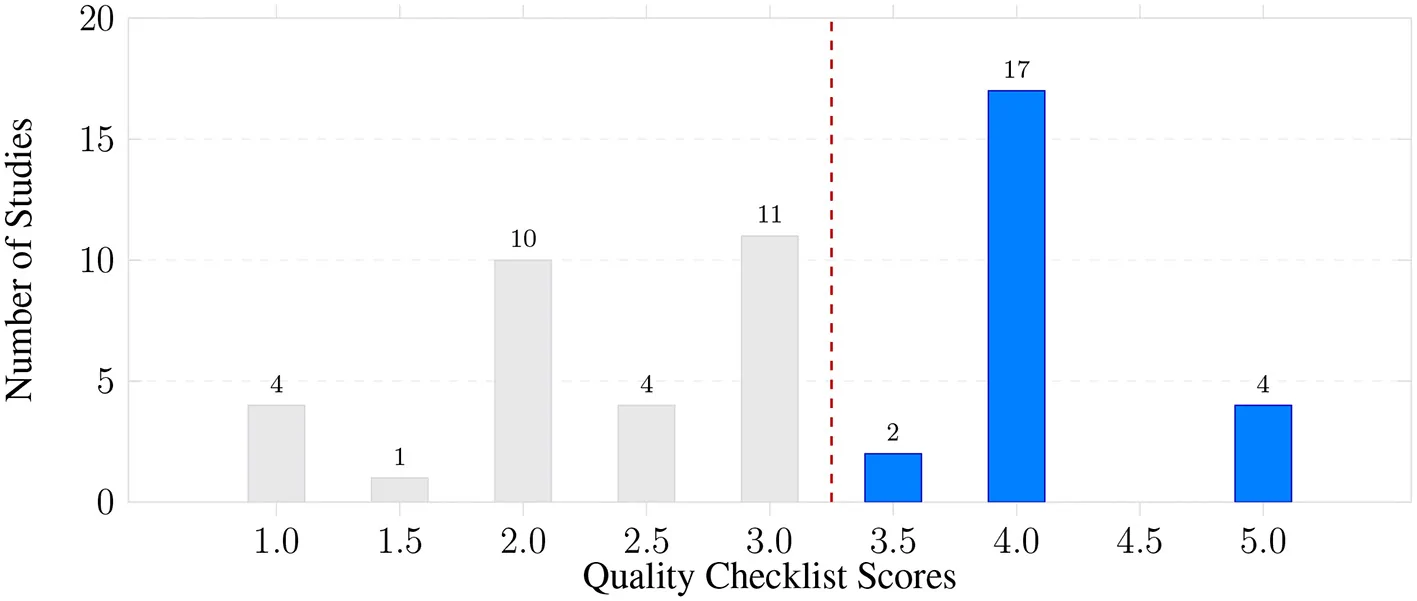
Number of studies by final score. Blue bars represent studies meeting the > 3.0 quality threshold.

To execute the second stage of filtering, the quality checklist presented in Section 2.5 was applied to the articles that passed the first selection stage. At the end of this stage, as a final result, of the 52 articles chosen in the previous stage, only 22 received a score of 3.0 points or higher on the checklist and were classified as Accepted. The remaining articles were classified as Rejected and excluded from the mapping process. These values can be seen in Figure 6. The number of studies and their respective final scores are presented in Figure 7.

## 4 Data synthesis and result presentation

**Figure 8:**
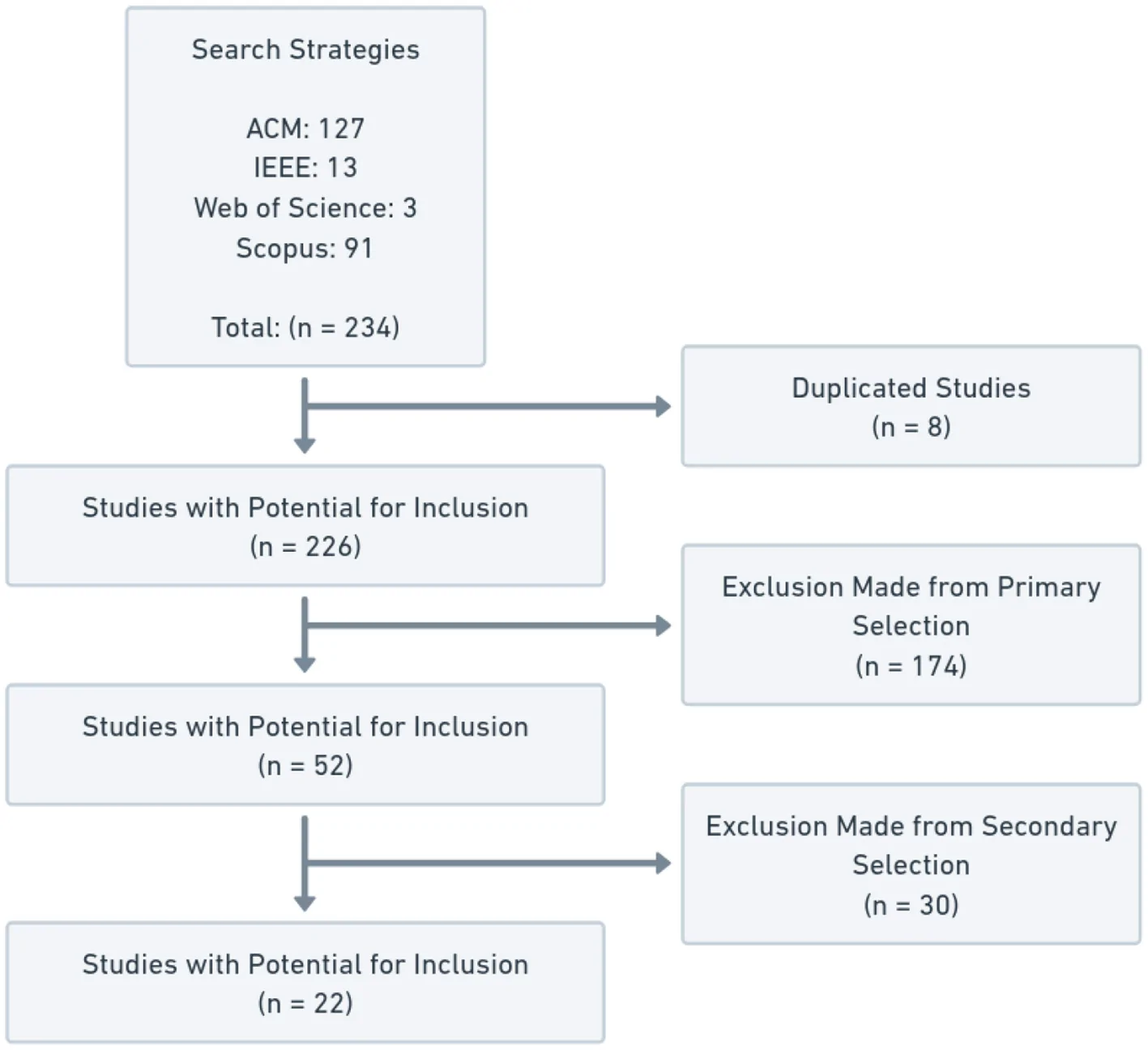
PRISMA diagram with data extraction.

In this section, the results of the systematic mapping are presented. Figure 8 shows a flowchart describing the extraction process of the articles obtained in each stage. Following this, the research questions are answered based on the extracted data.

### 4.1 What are the layers that compose a Big Data architecture for healthcare?

**Figure 9:**
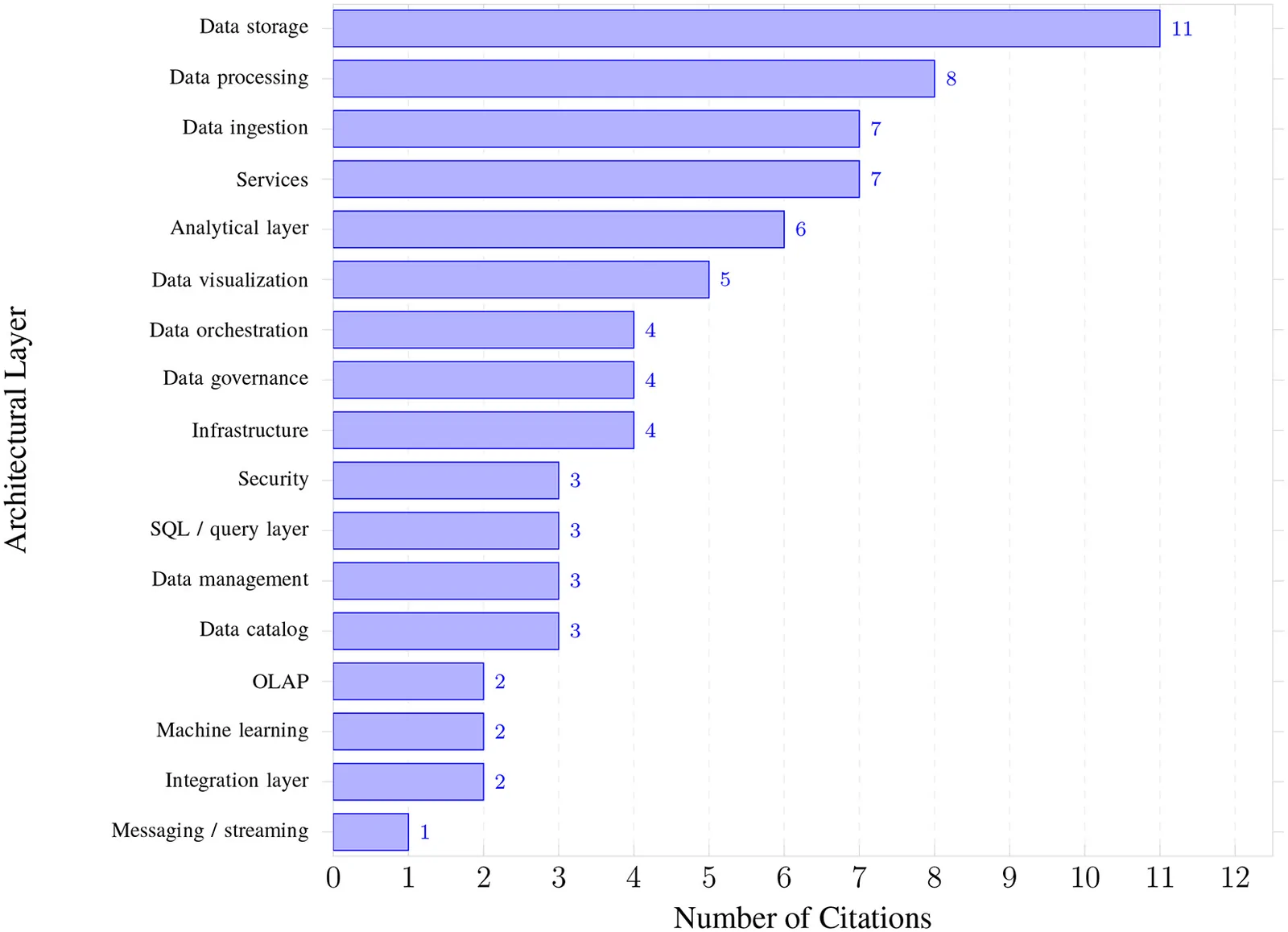
Architectural layers inferred from primary studies.

The analysis of the selected studies reveals a consensus on the fundamental layers of a Big Data architecture in healthcare, although terminology may vary among authors. As illustrated in Figure 9, the core functions of data storage, processing, and ingestion are consistently represented. The data storage layer was the most prominent, appearing in 21.15% of the articles, highlighting its central role and the challenges associated with scalability. This is followed by the data processing layer, present in 15.38% of the studies, and both data ingestion and service layers, each identified in 13.46% of the articles. Additionally, the analytical layer was found in 11.54% of the studies, while data visualization appeared in 9.62%. The remaining layers, including orchestration, governance, infrastructure, and security, were less frequently cited, each appearing in fewer than 8% of the studies. Table 5 details the specific layers mapped directly to the primary studies.

**Table 5:** Architectural layers present in the selected articles.

Architectural layer	Mapped citations
Data storage and indexing	(6, 12, 14, 17, 19, 20, 23, 51–55)
Data processing	(12, 14, 17, 19–21, 54, 55)
Services	(12, 19, 21, 23, 24, 54)
Data ingestion	(12, 19, 23, 53, 54)
Analytical applications	(13, 14, 23, 54, 56)
Data visualization	(6, 13, 23)
OLAP	(6, 13, 56)
Security	(24, 56)
SQL processing	(6, 12, 14)
Data catalog	(6, 13)
Resource provisioning	(17, 24)
Data and task orchestration	(13, 56)
Data management	(13, 14, 55)
Machine learning	(14, 56)
Data governance	(13, 56)

Interestingly, while there is consensus regarding the importance of the typical architectural layers, a significant gap remains, namely the absence of a dedicated **Statistical Integrity and Epidemiological Validation Layer**. The mapped studies predominantly reflect a computer science perspective, with a strong emphasis on data volume and processing speed. However, in the absence of a specific layer responsible for automated statistical pre checks, such as cohort balancing verification, anomaly detection, and epidemiological consistency validation, these architectures risk enabling a large scale “garbage in, garbage out” effect.

Therefore, as a **post-hoc** theoretical proposition derived from this identified gap, and explicitly distinct from the empirical findings of the mapped computer science literature, we argue that such a validation layer is necessary to adapt these general architectures to the demands of rigorous public health auditing environments.

### 4.2 Which software are the most used in big data architecture in healthcare?

A diverse set of software tools was identified across the selected studies, each addressing specific functionalities within the architectural layers. Table 6 summarizes the most cited software grouped by their architectural layer and function. In the data processing layer, tools like *Apache Spark* and *Hadoop MapReduce* are prevalent due to their robust capabilities for parallel and distributed computing. For real-time data streaming, *Apache Flink* and *Apache Spark* are frequently employed. For data ingestion, *Apache Kafka* is the preferred choice for managing real-time data flows. In the storage layer, while cloud-based solutions like *Amazon S3* are gaining traction, the trend in the reviewed literature leans toward open-source solutions that offer greater flexibility and cost-effectiveness. Finally, for data analysis and visualization, a combination of traditional Business Intelligence tools and modern platforms like *Elasticsearch* and *PrestoSQL* is common, enabling complex queries and real-time analytics to support clinical and operational decision-making. Furthermore, recent studies expand this open source ecosystem by highlighting tools such as *Apache Zookeeper* for cluster coordination and *Apache Cassandra* for highly available storage (12). For data pipeline automation, *dbt* (Data Build Tool) has emerged as a strong solution (13). Advanced querying and schema less discovery are frequently supported by *Apache Hive* and *Apache Drill* (14, 15), often relying on highly efficient storage formats like *Apache Parquet* (15, 16).

**Table 6:** Software and frameworks mapped to their respective architectural layers.

Architectural layer	Software, frameworks & tools
Data ingestion & messaging	Apache Kafka (6, 12, 19, 20, 22, 23, 54); Apache NiFi (19); Apache Sqoop (19); Apache Flume (54); Amazon Kinesis (6); Azure Queue System (53);
Data storage & databases	Apache Hadoop/HDFS (6, 12, 17, 19, 20, 22, 51, 53, 54, 57); Amazon S3 (6, 56); Google cloud storage (6); Ceph storage (17); Delta lake (23); MongoDB (22, 23, 55); Cassandra (54); Apache HBase (17, 51); PostgreSQL (23); Azure SQL server (53); Azure data warehouse (53); AWS Redshift (56); SciDB (13);
Data processing & analytics	Apache spark / spark streaming (6, 12, 17, 19, 20, 22, 23, 54); Apache Flink (6, 22); Apache Storm (19); Apache Pig (51); Hadoop-GIS (52); Apache Beam (6); Apache ADAM (14); Azure web jobs (53); Azure machine learning (53);
Querying, search & serving	Apache hive (17, 19, 51, 52); Elasticsearch (6, 19, 55); PrestoSQL (6); Apache AsterixDB (22); Apache Druid (6); Apache Pinot (6); Google BigQuery (56); SQL Server (14); MySQL (12); Apache drill (15);
Infrastructure, orchestration & management	Docker / docker swarm (17, 19); Kubernetes (17, 24); MetalLB (17); Apache Airflow (23); Apache Oozie (19); Apache Ambari (19); Apache Hadoop YARN (19); Apache ZooKeeper (19, 51); JupyterLab / JupyterHub (17); SCALPEL3 (58); REC ontology (23); dbt (13);

### 4.3 What comparisons between tools were presented in the study?

The primary studies presented several direct comparisons between tools to evaluate performance and efficiency. Rouzbeh et al. (17) demonstrated that querying data in the Optimized Row Columnar (ORC) format with Apache Hive (18) is significantly faster than using its original format. The same study also compared *Hadoop MapReduce* with *Apache Spark*, concluding that Spark's in-memory processing provides superior query speed by minimizing disk I/O. In another comparison, (6) found that *Apache Pinot* delivered query responses with 2 to 4 times lower latency than *Elasticsearch* when analyzing identical datasets. Regarding data storage efficiency, (19) compared various compression methods, finding that the “.avro” format combined with *snappy* compression yielded the best results, reducing storage footprint and improving data access speed. Expanding on these evaluations, (12) corroborated Spark performance advantage over traditional Hadoop and additionally identified *Apache Kafka* as superior to Flume due to better reliability and stream support. In the context of data pipelines, (13) showed that dbt provides significantly better automation, version control, and governance compared to traditional ETL methods. Architectural paradigms were also contrasted; (16) detailed how a Lakehouse architecture successfully merges the expansive storage of a Data Lake with the high performance of a Data Warehouse. Concurrently, (15) demonstrated that schema less methodologies offer immediate data access, overcoming the rigidity and delays inherent to traditional ETL. Lastly, regarding file formats, *Apache Parquet* was proven to drastically outperform CSV, JSON, and XML formats in both reading speed and compression (15, 16).

### 4.4 What are the fundamental aspects of a Big Data architecture for healthcare?

Beyond specific tools, the studies highlighted several fundamental concepts crucial for a successful Big Data architecture in healthcare. Data governance was a key theme, with (20) emphasizing the need for robust metadata management. The importance of real-time data flow was underscored by multiple authors; (21) and (22) both advocated for the use of message queues and pub/sub mechanisms for data streaming and fusion. Given the sensitive nature of health information, security is paramount. (17) pointed to the necessity of layered security and secure access through Virtual Private Networks (VPNs). On the data storage front, (23) introduced the use of modern table formats like Delta Lake, which provide high compression and efficient columnar storage. Finally, architectural design patterns were also discussed, with (24) recommending a microservices approach managed by container orchestrators like *Docker Swarm* (25) or *Kubernetes* (26), often fronted by an API Gateway to simplify service communication. Additionally, recent literature stresses the importance of parallel processing scalability and optimized cluster management to handle massive heterogeneous datasets (12, 15). Automation and strict version control in data transformation pipelines are now recognized as essential for maintaining continuous data quality (13). Modern systems also prioritize robust ACID transaction support, data freshness, and native readiness for AI and machine learning workloads (16). Moreover, the adoption of schema less flexibility is highly recommended to accelerate data discovery without demanding rigid upfront modeling (15).

Finally, an essential aspect largely missing from the strictly technical literature is methodological adherence to already existing medical protocols, such as the STROBE (Strengthening the Reporting of Observational Studies in Epidemiology) statement. Recent findings demonstrate that even massive datasets produce flawed clinical results if the architecture lacks a preliminary statistical and clinical consistency check. Works documenting phenomena such as “*Quantifying structural selection bias in observational cohort data…”* provide a concrete example of why this validation aspect is mandatory, proving that architectural scale does not replace methodological rigor (27).

### 4.5 In which years were articles most published in this field?

**Figure 10:**
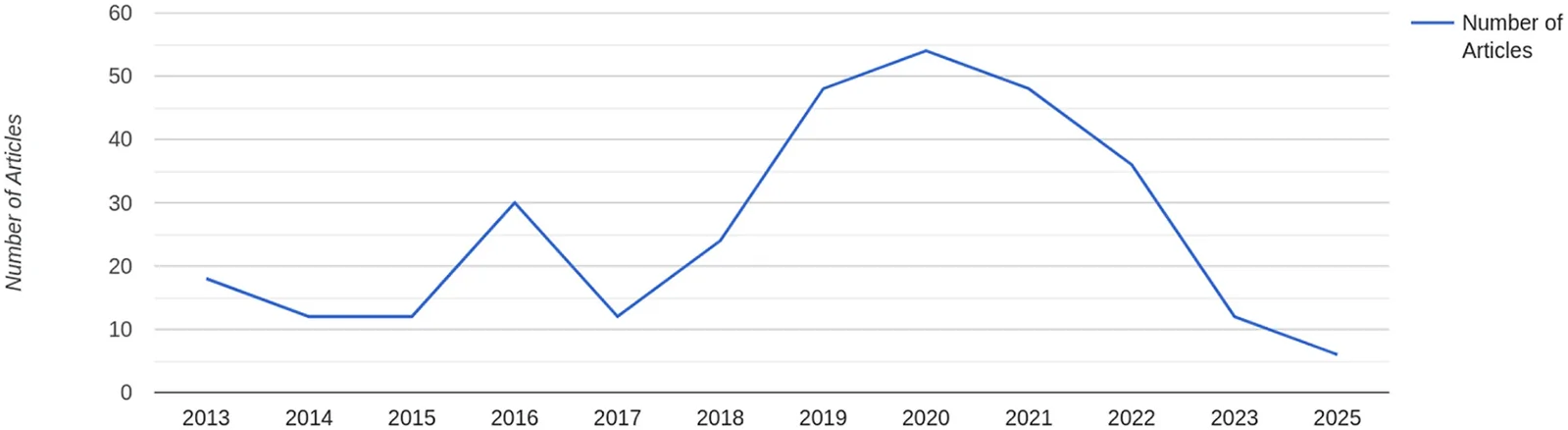
Selected studies by year of publication.

Figure 10 shows the selected studies ordered by publication year. It can be noted that most articles were written from 2019 to 2021.

### 4.6 What were the most popular methods of publication?

**Figure 11:**
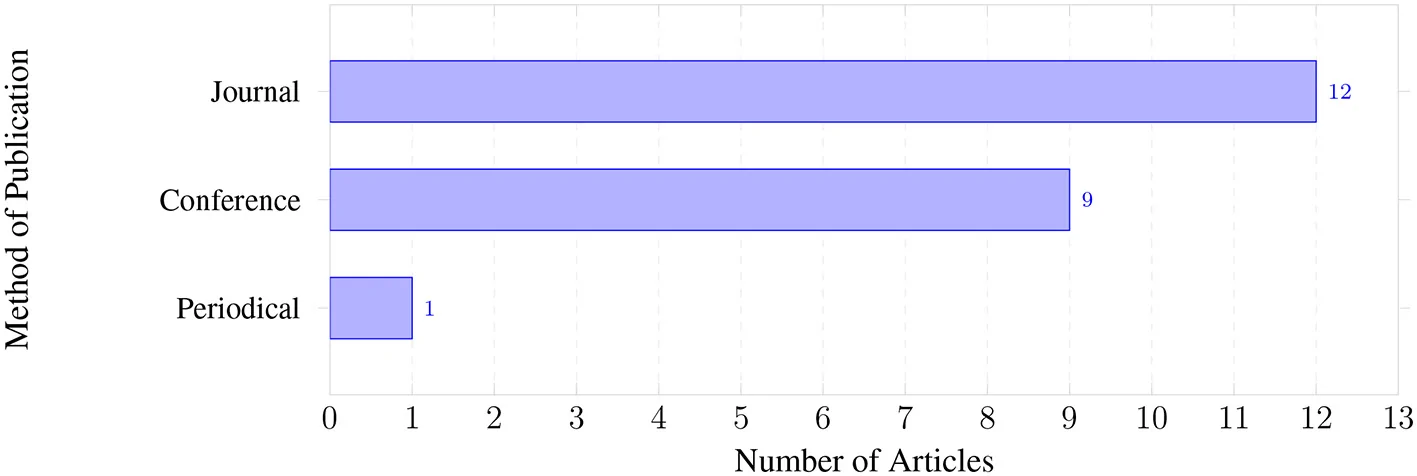
Number of articles by method of publication.

Figure 11 presents the selected studies by type of publication. Most studies were published in journals, with a total of 12 out of the 22 selected articles, representing 54.55% of the total. Conference papers account for 9 studies (40.91%), while only 1 study (4.55%) was classified as a periodical.

### 4.7 Which countries have most publications in this field?

**Figure 12:**
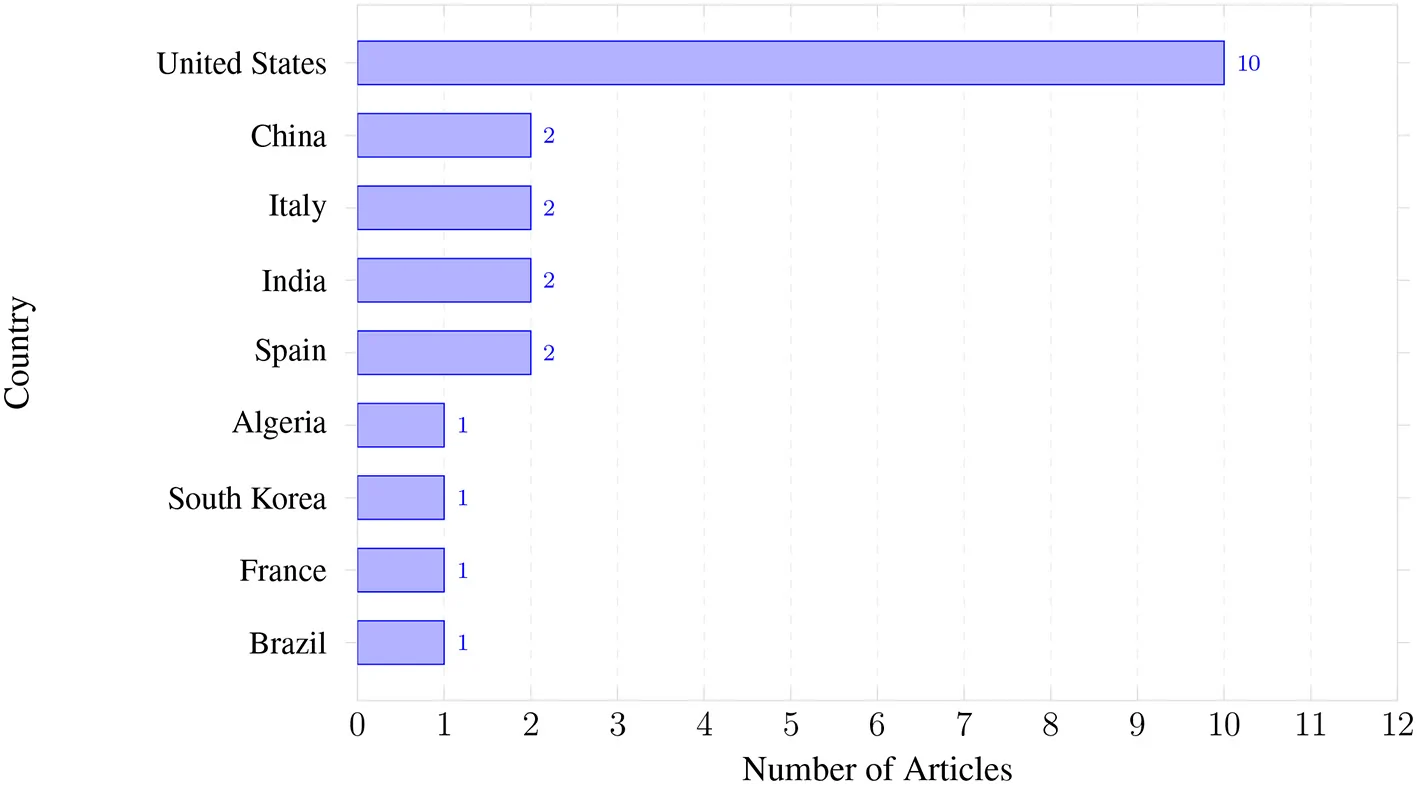
Number of articles by country of the first author.

Figure 12 presents the countries that most published about the field covered in this mapping. The country with the most publications is the United States, followed by Italy and Spain.

## 5 Narrative synthesis

### 5.1 Overview and key insights from the systematic mapping

The analysis of the selected studies highlights both the diversity and the fragmentation in the approaches to Big Data architectures for healthcare. While numerous architectures were presented, they often focus on specific subdomains of healthcare, with a lack of standardization across implementations. This diversity suggests that while there is no one-size-fits-all solution, the adaptability of architectures to meet specific needs is crucial. Notably, no architecture specifically targets auditing environments in healthcare, representing a significant opportunity. Instead of directly finding an auditing solution, this review maps the technological building blocks that can be *adapted* for this unmet need. It is crucial to emphasize that the empirical evidence gathered strictly reflects a computer science perspective; bridging this infrastructure to auditing requires the theoretical addition of clinical validation mechanisms not found in the primary studies.

The varying software and frameworks identified across the studies underline the absence of a universal solution. Different healthcare systems exhibit unique requirements, influencing their choice of architecture, layers, and tools. While the importance of scalability and performance is widely acknowledged, the integration of real-time data processing, advanced analytics, and data visualization appear to be key differentiators for the most advanced architectures.

Additionally, security and data governance are recurring themes, especially in the context of healthcare data privacy regulations. Several studies emphasize the need for robust security measures, including the use of VPNs and token-based access controls, underscoring the importance of maintaining the integrity and confidentiality of sensitive healthcare data.

Looking forward, the implementation of a Big Data architecture within the FiscalizaSUS platform, a collaboration between DenaSUS, LAIS, and NAVI, will leverage the best practices identified in this review. By adapting these general healthcare Big Data architectures for the specific unmet needs of auditing, the project aims to create a scalable platform that supports strategic planning and policy development while guaranteeing methodological integrity. This case study will serve as a concrete example of how Big Data architectures can be adapted to meet specific regulatory and operational needs, with the potential to guide future healthcare-related Big Data initiatives worldwide.

### 5.2 Emerging trends and technological innovations in big data architectures for healthcare infrastructure

The landscape of Big Data architectures for healthcare infrastructure is constantly changing, driven by technological advancements that seek to optimize performance, scalability, security, and system intelligence. This subsection explores the latest innovations, with an emphasis on advanced Artificial Intelligence (AI), new data management approaches, and software paradigms that are defining the future of digital health.

#### 5.2.1 Artificial intelligence (AI) and machine learning at the forefront of infrastructure

AI continues to be a primary driver of innovation, with new techniques and approaches being natively integrated into data architectures to enhance healthcare infrastructure.

1. **Operationalized Machine Learning (MLOps) and AIOps:** The application of MLOps, with tools like MLflow (28) and Kubeflow (29), is fundamental for managing the lifecycle of AI models. Furthermore, the concept of **AIOps (AI for IT Operations)** emerges, which uses AI to automate and optimize the management of the Big Data infrastructure itself, predicting failures, optimizing resources, and improving the resilience of healthcare systems (30).
2. **Natural Language Processing (NLP) and Generative AI:** Advanced language models such as BERT (31) and GPT-3 (32), and now their successors and specialized models like Med-PaLM 2 (33), are transforming the analysis of unstructured data in healthcare. However, Generative AI in healthcare is prone to flaws because it lacks the common sense of epidemiological boundaries. When trained on unverified healthcare data, there is a high risk of artifact amplification and hallucination propagation. Therefore, strict guardrails–such as Responsible AI principles, explainability requirements, and the aforementioned Statistical Integrity and Epidemiological Validation Layer–are mandatory to prevent AI from magnifying structural artifacts present in the raw data.
3. **Explainable AI (XAI) and Responsible AI:** The need for transparency in critical healthcare decisions drives XAI, with tools like SHAP (34) and LIME (35). The broader concept of **Responsible AI** also gains prominence, encompassing fairness, privacy, security, and interpretability, ensuring that AI solutions in healthcare infrastructure are ethical and trustworthy (36).
4. **Federated Learning:** Essential for healthcare due to privacy constraints and the distributed nature of data, Federated Learning allows AI models to be trained on multiple decentralized data sources (hospitals, clinics) without the raw data needing to be moved to a central location. This enhances privacy and security while enabling the creation of more robust and generalizable models (37). The infrastructure needs to be designed to support the secure orchestration and aggregation of these partial models.

#### 5.2.2 Evolutions in software and data management paradigms

New software approaches and data management strategies are crucial for handling the increasing complexity and volume of information in healthcare.

1. **Data Lakehouses on High-Performance Object Storage:** A foundational shift in data architecture involves moving from coupled systems like HDFS to a decoupled model based on object storage. While pioneered by cloud services like Amazon S3, this trend has been accelerated by the rise of high-performance, S3-compatible solutions like MinIO, which enable robust on-premise and hybrid cloud deployments (38). This decoupling of compute and storage allows for independent scaling, cost efficiency, and flexibility. On this foundation, the **Data Lakehouse** paradigm has emerged, merging the low-cost, scalable storage of data lakes with the performance and transactional guarantees of data warehouses. This is achieved through open table formats like **Delta Lake** (39), **Apache Iceberg** (40), and **Apache Hudi** (41), which provide ACID transactions, data versioning (time travel), and schema enforcement directly on data in object stores. For healthcare, this means large volumes of structured and unstructured data can be managed reliably and analyzed with high performance.
2. **Vector Databases for AI:** With the proliferation of AI-generated embeddings (for text, images, genomic data), vector databases such as Pinecone (42), Milvus (43), and Weaviate (44) become essential infrastructure components. They are optimized for high-speed similarity searches, fundamental for applications like image-assisted diagnosis, semantic search in medical records, and personalized medicine.
3. **Confidential Computing and Privacy Preservation:** Beyond encryption in transit and at rest, **Confidential Computing** (using Trusted Execution Environments - TEEs) and advanced encryption techniques like **Partial/Fully Homomorphic Encryption** are maturing. They allow the processing of sensitive health data while it is still encrypted or within secure enclaves, opening doors for secure collaborations without exposing raw data (45).
4. **Streaming Platforms and Event-Driven Architectures:** Real-time data streaming platforms like Apache Pulsar (46) and Redpanda (47) are vital. The adoption of **Event-Driven Architectures (EDA)** in healthcare allows systems to react dynamically to significant events (new test results, patient monitoring alerts), facilitating interoperability and real-time decision-making.
5. **Data Mesh as an Organizational and Technical Paradigm:** Data Mesh proposes a paradigm shift, decentralizing data ownership and architecture. Instead of a centralized data lake or data warehouse, data is treated as a product, owned by specific business domains (e.g., radiology department, inpatient unit). This promotes agility, scalability, and greater alignment with business needs in healthcare infrastructure, although it requires robust federated governance (48).
6. **Edge Computing and IoT in Healthcare:** The proliferation of connected medical devices (IoT) and the need for low latency for critical applications (such as real-time monitoring and robot-assisted surgery) drive **Edge Computing**. Processing data closer to where it is generated reduces latency, saves bandwidth, and can improve privacy by minimizing raw data transfer. Big Data infrastructure needs to extend to the edge to efficiently collect, process, and analyze this data (49).

## 6 Threats to validity

Threats to validity refer to factors that may limit the ability to correctly interpret or generalize the results obtained in the systematic review (50). In the context of this study, some threats must be considered to ensure the robustness of the conclusions.

**Construct validity**

1. **Search strategy and methodological blindness:** As noted in the methodology, the search strategy predominantly targeted Computer Science terminology. By excluding terms specifically related to “Data Veracity” or “Epidemiological Standards”, the survey risks a phenomenon we term “Methodological Blindness”. A review that maps technologies but ignores the epidemiological quality and validation of the data being moved is inherently biased toward a purely engineering perspective. This represents a threat to its validity in a Public Health context, where data integrity is as critical as processing speed. To mitigate this threat in general terms, a comprehensive and iterative search process was employed, including the use of multiple databases and keywords refined through expert feedback and control articles identified via the PICO model. Additionally, the inclusion and exclusion criteria were specifically designed to balance the scope of the study with the relevance and quality of the selected articles.

**Internal validity**

1. **Selection bias:** The criteria used to select studies may introduce bias if the included articles do not adequately represent the full spectrum of healthcare Big Data architectures. To minimize this risk, studies were selected based on clear and transparent criteria, which were applied consistently by the research team.

**External validity**

1. **Data synthesis limitations and indexing behaviors:** The synthesis of data from the selected studies may be influenced by variations in methodology, terminology, and focus areas across the articles. To mitigate this, the analysis was designed to identify common trends and key findings. However, the diversity in the architectures and software tools used in the studies may make it difficult to draw direct comparisons. Furthermore, database-specific indexing behaviors can introduce selection bias. For instance, the highly restrictive intersection of computer science performance terminology with specific healthcare domains in our search string yielded proportionally lower initial results in databases like Web of Science (n=3) and IEEE Xplore (n=13) compared to ACM. While the final sample size of 22 primary studies could limit the broadest generalizability, this specific corpus is a direct, methodologically sound result of the stringent inclusion/exclusion criteria focused specifically on architectural performance and scalability.

## 7 Conclusion

This study conducted a systematic mapping to identify and characterize the approaches, concepts, and software used in building a general *Big Data* architecture for healthcare, with a particular focus on performance and scalability. A total of 234 studies were identified, from which 22 were selected after applying inclusion and exclusion criteria, alongside a quality evaluation. The majority of the selected articles were published between 2019 and 2021. Journals remained the predominant publication venue, accounting for 12 studies (54.55%) of the total. Conference papers represented 9 studies (40.91%), while only 1 study (4.55%) was classified as a periodical. Geographically, the United States emerged as the leading country in terms of publications.

Most studies addressed the primary layers of a *Big Data* architecture. Among these, the data storage, data ingestion, data processing, and service layers were the most frequently discussed. This indicates a broad consensus on the fundamental components of *Big Data* systems in healthcare, although variations in terminology and focus were observed.

Crucially, the review found no architecture specifically designed for healthcare auditing systems. Therefore, the core contribution of this work lies in outlining the technological building blocks that must be *adapted* to meet this unmet need. Projects such as the *FiscalizaSUS* platform in Brazil can leverage these findings by transitioning from legacy frameworks to modern, decoupled cloud-native solutions.

The software solutions highlighted in the studies aim to address the specific needs of each layer of the *Big Data* architecture. Among the most mentioned tools, *Hadoop HDFS* stood out for data storage, *Apache Spark* for data processing, *Apache Kafka* for data ingestion, and *Elasticsearch* for data search and visualization. These tools represent a strong trend toward open-source solutions, offering scalability, flexibility, and cost-efficiency for healthcare organizations.

Several studies also provided comparisons between software tools and explored key concepts such as data compression and latency reduction. For instance, the use of columnar formats like ORC files was shown to improve query performance while reducing storage requirements. Additionally, some tools demonstrated lower latency in query responses compared to others, highlighting the importance of selecting the right tool for specific performance needs in healthcare environments. Furthermore, paradigm shifts such as the *Lakehouse* architecture and *schema-less* querying methodologies proved to offer significant improvements over traditional Data Warehouse and ETL rigidities.

Security and access control emerged as crucial considerations when designing *Big Data* architectures for healthcare. Concepts like secure network access via VPN and the implementation of microservices orchestrated by tools like *Docker* and *Kubernetes* were frequently mentioned as best practices for ensuring data security and system scalability. Beyond infrastructure, current fundamentals also strongly emphasize data governance through version control, robust ACID transaction support, data freshness, and architectures natively ready to process Artificial Intelligence and Machine Learning workloads directly on the stored data.

Finally, the most critical realization for digital public health is that engineering performance and scalability are insufficient on their own. The widespread absence of a dedicated Statistical Integrity and Epidemiological Validation Layer in the mapped literature is a major flaw. To address this, we advance the concept of this validation layer not as an empirical result of this mapping, but as a mandatory post-hoc theoretical proposal. Future healthcare Big Data initiatives, particularly in auditing, must prioritize automated epidemiological and clinical consistency checks to avoid processing biased data at high speeds. True advancement in this field requires aligning technical scalability with rigorous scientific validation. Continuous vigilance and adaptation to these technological and methodological novelties will be determinant for the success and sustainability of Big Data initiatives in healthcare. As a next step, leveraging these insights, the development of a *Big Data* architecture tailored for auditing Brazil's healthcare system is suggested, providing an opportunity to apply the knowledge gathered in this study to a concrete, high-impact project.

## Data Availability

The original contributions presented in the study are included in the article/supplementary material, further inquiries can be directed to the corresponding authors.

## References

[1] Stylianou A Talias MA. Big data in healthcare: a discussion on the big challenges. Health Technol. (2016) 7:97–107. doi: 10.1007/s12553-016-0152-4

[2] Wu X Zhu X Wu GQ Ding W. Data mining with big data. IEEE Trans Knowl Data Eng. (2014) 26:97–107. doi: 10.1109/TKDE.2013.109

[3] Carvalho D Cruz R. Big data and machine learning in health. Eur J Public Health. (2020) 06:30. doi: 10.1093/eurpub/ckaa040.030

[4] Parhami B. Parallel Processing with Big Data. Cham: Springer International Publishing (2018). p. 1–7.

[5] Ramesh B. In: Mohanty H, Bhuyan P, Chenthati D. Big Data Architecture. New Delhi: Springer India (2015). p. 29–59.

[6] Fu Y Soman C. Real-time Data Infrastructure at Uber. CoRR abs/2104.00087. (2021). Available online at: https://arxiv.org/abs/2104.00087 (Accessed May 15, 2025).

[7] Kitchenham B. Procedures for Performing Systematic Reviews. Keele: Keele Univ. (2004).

[8] Petersen K Vakkalanka S Kuzniarz L. Guidelines for conducting systematic mapping studies in software engineering: an update. Inform Softw Technol. (2015) 64:1–18. doi: 10.1016/j.infsof.2015.03.007

[9] Santos CMdC Pimenta CAdM Nobre MRC. The PICO strategy for the research question construction and evidence search. Revista Latino-Americana de Enfermagem. (2007) 15:508–11. doi: 10.1590/S0104-11692007000300023 17653438

[10] Capes. Portal de periódicos CAPES/MEC [Journal Portal CAPES/MEC]. (2023). Available online at: https://www.periodicos.capes.gov.br (accessed June 01, 2023).

[11] Scopus. Scopus - *Elsevier Database*. (2023). Available online at:https://www.scopus.com/ (accessed June 01, 2023).

[12] Tu Y Lu Y Chen G Zhao J Yi F. Architecture design of distributed medical big data platform based on spark. In: 2019 IEEE 8th Joint International Information Technology and Artificial Intelligence Conference (ITAIC). Chongqing: IEEE. (2019). p. 682–685.

[13] Murugan AS Saravanan SK Nidhya GS Sasikala K Pandey P Arasan E. Leveraging data build tool for efficient data management in data center architectures. In: 2025 11th International Conference on Communication and Signal Processing (ICCSP). Melmaruvathur: IEEE (2025). p. 502–507.

[14] Begoli E Kistler D Bates J. Towards a heterogeneous, polystore-like data architecture for the US Department of Veteran Affairs (VA) enterprise analytics. In: 2016 IEEE International Conference on Big Data (Big Data). Washington, DC: IEEE. (2016). p. 2550–2554. doi: 10.1109/BigData.2016.7840896

[15] Begoli E Dunning T Frasure C. Real-time discovery services over large, heterogeneous and complex healthcare datasets using schema-less, column-oriented methods. In: 2016 IEEE Second International Conference on Big Data Computing Service and Applications (BigDataService). Oxford: IEEE (2016). p. 257–264.

[16] Begoli E Goethert I Knight K. A lakehouse architecture for the management and analysis of heterogeneous data for biomedical research and mega-biobanks. In: 2021 IEEE International Conference on Big Data (Big Data). Orlando, FL: IEEE. (2021). p. 4643–4651.

[17] Rouzbeh F Grama A Griffin PM Adibuzzaman M. Collaborative cloud computing framework for health data with open source technologies. CoRR. abs/2007.10498. (2020). Available online at: https://arxiv.org/abs/2007.10498.

[18] Foundation TAS. Apache Hive Documentation (2024). Available online at: https://hive.apache.org/ (Accessed May 15, 2025).

[19] McPadden J Durant TJ Bunch DR Coppi A Price N Rodgerson K . Health care and precision medicine research: analysis of a scalable data science platform. J Med Internet Res. (2019) 21:e13043. doi: 10.2196/13043 30964441 PMC6477571

[20] Chen HM Kazman R Haziyev S Hrytsay O. Big data system development: an embedded case study with a global outsourcing firm. In: 2015 IEEE/ACM 1st International Workshop on Big Data Software Engineering (2015). p. 44–50.

[21] Ahmad A Paul A Rathore M Chang H. An efficient multidimensional big data fusion approach in machine-to-machine communication. ACM Trans Embed Comput Syst. (2016) 15:2834118. doi: 10.1145/2834118

[22] Jacobs S Wang X Carey MJ Tsotras VJ Uddin MYS. BAD to the Bone: Big Active Data at its Core. CoRR abs/2002.09755. (2020). Available online at: https://arxiv.org/abs/2002.09755 (Accessed May 15, 2025).

[23] Gagliardelli L Zecchini L Ferretti L Beneventano D Simonini G Bergamaschi S . A big data platform exploiting auditable tokenization to promote good practices inside local energy communities. Future Gener Comput Syst. (2023) 141:595–610. doi: 10.1016/j.future.2022.12.007

[24] Calderón-Gómez H Mendoza-PittíL Vargas-Lombardo M Gómez-Pulido JM Rodríguez-Puyol D Sención G . Evaluating service-oriented and microservice architecture patterns to deploy eHealth applications in cloud computing environment. Appl Sci. (2021) 11:4350. doi: 10.3390/app11104350

[25] Inc D. Docker Swarm Documentation. (2024). Available online at: https://docs.docker.com/engine/swarm/ (Accessed May 15, 2025).

[26] Llc G. Kubernetes Documentation. (2024). Available online at: ttps://kubernetes.io (Accessed May 15, 2025).

[27] Roccetti M. Quantifying structural selection bias in observational cohort data: a ponderation analysis of age specific incidence rates to inform vaccine safety verification. Front Pharmacol. (2026) 16:1754809. doi: 10.3389/fphar.2025.1754809 41585872 PMC12824001

[28] Zaharia M Chen A Davidson A Ghodsi A Hong SA Liang E . Accelerating the machine learning lifecycle with MLflow. In: Proceedings of the 4th MLSys Conference. (2018).

[29] Kubeflow. Kubeflow - *Machine Learning Toolkit for Kubernetes*. (2018). Available online at: https://www.kubeflow.org/ (accessed May 13, 2025).

[30] Gartner Inc. Market Guide for AIOps Platforms; 2022. Acessado em [coloque a data de acesso]. Disponível em: [Se possível, link para o sumário público ou relatório, caso contrário, citar como relatório da Gartner]. Relatório da Gartner. Stamford, CT: Gartner Inc.

[31] Devlin J Chang MW Lee K Toutanova K. BERT: pre-training of deep bidirectional transformers for language understanding. arXiv [preprint] arXiv:181004805. (2018). doi: 10.48550/arXiv.1810.04805

[32] Brown TB Mann B Ryder N Subbiah M Kaplan J Dhariwal P . Language models are few-shot learners. arXiv [preprint] arXiv:200514165. (2020). doi: 10.48550/arXiv.2005.14165

[33] Singhal K Azizi S Tu T Mahdavi SS Wei J Chung HW . Large language models encode clinical knowledge. Nature. (2023) 620:172–80. doi: 10.1038/s41586-023-06291-2 37438534 PMC10396962

[34] Lundberg SM Lee SI. A unified approach to interpreting model predictions. In: Proceedings of the 31st International Conference on Neural Information Processing Systems (2017). p. 4765–4774.

[35] Ribeiro MT Singh S Guestrin C. “Why should i trust you?” Explaining the predictions of any classifier. In: Proceedings of the 22nd ACM SIGKDD International Conference on Knowledge Discovery and Data Mining. New York: ACM (2016). p. 1135–1144.

[36] Floridi L Cowls J Beltramini M Saunders D Vayena E. The ethical framework for a good AI society: opportunities, risks, principles, and recommendations. AI and Society. (2021) 36:689–707. doi: 10.1007/s11023-018-9482-5 30930541 PMC6404626

[37] Rieke N Hancox J Li W. MilletarìF, Roth HR, Albarqouni S, et al. The future of digital health with federated learning. NPJ Digit Med. (2020) 3:119. doi: 10.1038/s41746-020-00323-1 33015372 PMC7490367

[38] Adar E Gerhards L Grunzke R Hartmann V Henkel R Schlarb M . Performance evaluation of an S3-compatible object storage for scientific data. In: 2022 IEEE/ACM 7th International Workshop on Data-driven User-centric Systems and Log Analysis (DUST). (2022). p. 1–10.

[39] Armbrust M Das T Ghodsi A Interlandi M Luszczyk F Murthy A . Delta lake: high-performance ACID table storage over cloud object stores. In: Proceedings of the 46th International Conference on Very Large Data Bases. California: VLDB Endowment (2020). p. 3411–3424.

[40] Jain P Kraft P Power C Das T Stoica I Zaharia M. Analyzing and comparing lakehouse storage systems. In: Proceedings of the 13th Annual Conference on Innovative Data Systems Research (CIDR 23). Santa Cruz, CA: CIDR Association (2023).

[41] The Apache Hudi Project. Apache Hudi: Upserts and Incremental Processing on Big Data (2016). Available online at: https://hudi.apache.org/ (accessed May 14, 2025).

[42] Pinecone Systems Inc. Pinecone Documentation. (2025). https://docs.pinecone.io (Accessed May 19, 2025).

[43] Wang Z Feng Y Li L Zhou W Li J Wang R . Milvus: a purpose-built vector data management system. Proc VLDB Endowment. (2021) 14:2190–201. doi: 10.1145/3448016.3457550

[44] Weaviate Team. Weaviate - *The Open Source Vector Database*. Amsterdam: Weaviate B.V. (2024). Available online at: https://weaviate.io/ (Accessed May 15, 2025).

[45] Roumpies F Kakarountas A. A Review of Homomorphic Encryption and its Contribution to the Sector of Health Services. doi: 10.1145/3635059.3635096

[46] Jain V Ahuja A Saini D. Evaluation and Performance Analysis of Apache Pulsar and NATS. Singapore: Springer Nature Singapore (2022). p. 179–190.

[47] Akidau T Bradshaw R Chambers C Chernyak R Lax R Chernyak S . The dataflow model: a practical approach to balancing correctness, latency, and cost in massive-scale, out-of-order data processing. Proc VLDB Endowm. (2015) 8:1792–803. doi: 10.14778/2824032.2824076

[48] Dehghani Z. Data mesh: delivering data-driven value at scale. Sebastopol: O'Reilly Media. (2022).

[49] Safaei M. Edge Computing Applications for IoT in Healthcare: A Systematic Literature Review. doi: 10.21203/RS.3.RS-690045/V1

[50] Chapetta WA. Uma infra-estrutura para planejamento, execuçã e empacotamento de estudos experimentais em engenharia de software. UFRJ. Rio de Janeiro: Federal University of Rio de Janeiro (COPPE/UFRJ) (2006).

[51] Sebaa A Nouicer A Chikh F Tari A. Big data technologies to improve medical data warehousing. In: Proceedings of the 2nd International Conference on Big Data, Cloud and Applications BDCA'17. New York, NY: Association for Computing Machinery. (2017).

[52] Aji A Sun X Vo H Liu Q Lee R Zhang X . Demonstration of Hadoop-GIS: a spatial data warehousing system over MapReduce. In: Proceedings of the 21st ACM SIGSPATIAL International Conference on Advances in Geographic Information Systems. SIGSPATIAL'13. New York, NY, USA: Association for Computing Machinery (2013). p. 528–531. 10.1145/2525314.2525320 PMC5013659 27617325

[53] RubíJNS Gondim PRL. IoMT platform for pervasive healthcare data aggregation, processing, and sharing based on OneM2M and OpenEHR. Sensors. (2019) 19:4283. doi: 10.3390/s19194283 31623304 PMC6806104

[54] Alonso-Gonzqález CJ Pulido B Cartón M Bregon A. A big data architecture for fault prognostics of electronic devices: application to power MOSFETs. IEEE Access. (2019) 7:102160–73. doi: 10.1109/ACCESS.2019.2929111

[55] Delussu G Lianas L Frexia F Zanetti G. A scalable data access layer to manage structured heterogeneous biomedical data. PLoS ONE. (2016) 11:1–38. doi: 10.1101/067371 27936191 PMC5148592

[56] Nambiar A Mundra D. An overview of data warehouse and data lake in modern enterprise data management. Big Data Cognit Comp. (2022) 6:132. doi: 10.3390/bdcc6040132

[57] Bohlouli M He Z. EMR: scalable clustering of big HR data using evolutionary MapReduce. In: Companion Proceedings of the Web Conference 2021, WWW '21. New York, NY: Association for Computing Machinery (2021). p. 26–34.

[58] Bacry E Gaïffas S Leroy F Morel M Nguyen DP Sebiat Y . SCALPEL3: A scalable open-source library for healthcare claims databases. Int J Med Inform. (2020) 141:104203. doi: 10.1016/j.ijmedinf.2020.104203 32485553

