# Correction: Scientific and technological mapping of big data architectures in healthcare

**DOI:** 10.3389/fpubh.2026.1945661

**Published:** 2026-08-24

**Authors:** Helder Prado Santos, Methanias Colaço Júnior, Ricardo Valentim, João Paulo Queiroz dos Santos, Marcia Elizabeth Marinho da Silva, Luiz Affonso Henderson Guedes de Oliveira, Guilherme Medeiros Machado, Antonio Higor Freire de Morais, Marianne Dantas Farias Vieira, Deborah Dantas Arruda, Gleyson José Pinheiro Caldeira Silva, Raphael Silva Fontes

**Affiliations:** 1 Laboratory for Technological Innovation in Health (LAIS), Onofre Lopes University Hospital, Federal University of Rio Grande do Norte (UFRN), Natal, Rio Grande do Norte, Brazil; 2 Center for Innovation and Advanced Technology (NAVI), Federal Institute of Rio Grande do Norte (IFRN), Natal, Rio Grande do Norte, Brazil; 3 Postgraduate Program in Computer Science (PROCC), Federal University of Sergipe, São Cristóvão, Brazil; 4 Brazilian Ministry of Health, Brasília, Brazil; 5 ECE Paris Engineering School, Paris, France

**Keywords:** architecture, big data, data lake, health, performance, public health auditing, scalability

Affiliations “Postgraduate Program in Computer Science (PROCC), Federal University of Sergipe, São Cristóvão, Brazil”, and “Center for Innovation and Advanced Technology (NAVI), Federal Institute of Rio Grande do Norte, Natal, Brazil” were erroneously omitted for author Helder Prado Santos.

Affiliations “Postgraduate Program in Computer Science (PROCC), Federal University of Sergipe, São Cristóvão, Brazil”, and “Center for Innovation and Advanced Technology (NAVI), Federal Institute of Rio Grande do Norte, Natal, Brazil” were erroneously omitted for author Methanias Colaço Júnior.

Affiliation “Laboratory for Technological Innovation in Health (LAIS), Onofre Lopes University Hospital, Federal University of Rio Grande do Norte, Natal, Brazil” was erroneously omitted for author Ricardo Valentim.

Affiliation “Computer Science, State University of Sergipe, Aracaju, Sergipe, Brazil” was erroneously included in the paper for author Ricardo Valentim. This affiliation has now been removed for author Ricardo Valentim.

Affiliation “Laboratory for Technological Innovation in Health (LAIS), Onofre Lopes University Hospital, Federal University of Rio Grande do Norte (UFRN), Natal, Rio Grande do Norte, Brazil” was erroneously included in the paper for author João Paulo Queiroz dos Santos. This affiliation has now been removed for author João Paulo Queiroz dos Santos.

Affiliation “Brazilian Ministry of Health, Brasília, Brazil” was erroneously omitted for author Marcia Elizabeth Marinho da Silva.

Affiliation “Laboratory for Technological Innovation in Health (LAIS), Onofre Lopes University Hospital, Federal University of Rio Grande do Norte (UFRN), Natal, Rio Grande do Norte, Brazil” was erroneously included in the paper for author Marcia Elizabeth Marinho da Silva. This affiliation has now been removed for author Marcia Elizabeth Marinho da Silva.

Affiliation “ECE Paris Engineering School, Paris, France” was erroneously omitted for author Guilherme Medeiros Machado.

Affiliation “Laboratory for Technological Innovation in Health (LAIS), Onofre Lopes University Hospital, Federal University of Rio Grande do Norte (UFRN), Natal, Rio Grande do Norte, Brazil” was erroneously included in the paper for author Guilherme Medeiros Machado. This affiliation has now been removed for author Guilherme Medeiros Machado.

Affiliation “Center for Innovation and Advanced Technology (NAVI), Federal Institute of Rio Grande do Norte, Natal, Brazil” was erroneously omitted for author Antonio Higor Freire de Morais.

Affiliation “Laboratory for Technological Innovation in Health (LAIS), Onofre Lopes University Hospital, Federal University of Rio Grande do Norte (UFRN), Natal, Rio Grande do Norte, Brazil” was erroneously included in the paper for author Antonio Higor Freire de Morais. This affiliation has now been removed for author Antonio Higor Freire de Morais.

Affiliation “Center for Innovation and Advanced Technology (NAVI), Federal Institute of Rio Grande do Norte, Natal, Brazil” was erroneously omitted for author Gleyson José Pinheiro Caldeira Silva.

Affiliation “Laboratory for Technological Innovation in Health (LAIS), Onofre Lopes University Hospital, Federal University of Rio Grande do Norte (UFRN), Natal, Rio Grande do Norte, Brazil” was erroneously included in the paper for author Gleyson José Pinheiro Caldeira Silva. This affiliation has now been removed for author Gleyson José Pinheiro Caldeira Silva.

Affiliation “Center for Innovation and Advanced Technology (NAVI), Federal Institute of Rio Grande do Norte, Natal, Brazil” was erroneously omitted for author Raphael Silva Fontes.

Affiliation “Laboratory for Technological Innovation in Health (LAIS), Onofre Lopes University Hospital, Federal University of Rio Grande do Norte (UFRN), Natal, Rio Grande do Norte, Brazil” was erroneously included in the paper for author Raphael Silva Fontes. This affiliation has now been removed for author Raphael Silva Fontes.

In **4 Data synthesis and result presentation**, *4.1 What are the layers that compose a Big Data architecture for healthcare?*, Paragraph 4 the following residual Portuguese text was inadvertently included in the published article during the production process and has been removed:

“Aqui estão as seções com os complementos adicionados, mantendo o texto original intacto exatamente como solicitado, evitando adicionar novos hifens nas inserções e utilizando as referências fornecidas para integrar as informações da tabela.”

The Funding statement was incorrect in the published version.

The correct **Funding** statement is:

The author(s) declared that financial support was received for this work and/or its publication. The publication fees were funded by the Laboratory for Technological Innovation in Health (LAIS), Onofre Lopes University Hospital, Federal University of Rio Grande do Norte (UFRN), Natal, Brazil.

The original version of this article has been updated.

